# Association of Single Nucleotide Polymorphisms in *LEP*, *LEPR,* and *PPARG* With Humoral Immune Response to Influenza Vaccine

**DOI:** 10.3389/fgene.2021.725538

**Published:** 2021-10-22

**Authors:** Mao Li, Hejiang Wei, Shuyi Zhong, Yanhui Cheng, Simin Wen, Dayan Wang, Yuelong Shu

**Affiliations:** ^1^ School of Public Health (Shenzhen), Shenzhen Campus of Sun Yat-sen University, Guangzhou, China; ^2^ National Institute for Viral Disease Control and Prevention, Chinese Center for Disease Prevention and Control, Beijing, China

**Keywords:** influenza vaccine, single nucleotide polymorphism (SNP), *LEP*, *LEPR*, *PPARG*, immune response

## Abstract

**Background:** Although previous studies have proposed leptin plays an important role in energy metabolism as well as in immune response, the effects of leptin-related genes on influenza vaccine-induced immune response remain unexplored. In this study, we aimed to investigate the potential association of leptin gene (*LEP*), leptin receptor gene (*LEPR*), and peroxisome proliferator activated receptor gamma gene (*PPARG*) polymorphisms with humoral immune response to influenza vaccine.

**Methods:** Based on the seroconversion to influenza vaccine, 227 low-responders and 365 responders were selected in this study, and 11 candidate single nucleotide polymorphisms (SNPs) were genotyped using the MassARRAY technology platform. Univariate and multivariate logistic regression analyses were used to explore the association of SNPs in *LEP*, *LEPR,* and *PPARG* with humoral immune response to influenza vaccine. We also conducted a stratified analysis by gender to further clarify this association. The haplotypes analysis was performed using SNPStats.

**Results:** Significant differences were observed in the genotypic distribution of *PPARG* rs17793951 between the two groups (*p* = 0.001), and the *PPARG* rs17793951 AG + GG genotype was associated with a higher risk of low responsiveness to influenza vaccine adjusted for gender and age (additive genetic model: OR = 2.94, 95% CI = 1.67–5.19, dominant genetic model: OR = 2.81, 95% CI = 1.61–4.92). No significant association of other SNPs in *LEP* and *LEPR* with immune response to influenza vaccine was found. The stratified analysis found the gender difference in the association of *LEPR* and *PPARG* variants with immune response to influenza vaccine. We found that *LEPR* rs6673591 GA + AA genotype was correlated with low responsiveness to influenza vaccine only in males (OR = 1.96, 95% CI = 1.05–3.67), and *PPARG* rs17793951 AG + GG genotype was associated with low responsiveness to influenza vaccine in females (OR = 3.28, 95% CI = 1.61–6.67). Compared with the CGGAGGC haplotype composed of *LEPR* rs1327118, rs7602, rs1137101, rs1938489, rs6673591, rs1137100, and rs13306523, the CAAAAAC haplotype was positively correlated with immune response of influenza vaccine (OR = 0.34, 95% CI = 0.15–0.77). Haplotype TG comprised of *PPARG* rs796313 and rs17793951 was associated with a 2.85-fold increased risk of low responsiveness to influenza vaccine.

**Conclusion:** Our study identified that *PPARG* rs17793951 variants were significantly associated with the immune response to influenza vaccine.

## Introduction

Influenza is an infectious respiratory disease transmitted by droplets or human-to-human close contacts. It is estimated that the seasonal influenza epidemic causes one billion cases worldwide every year, including three to five million severe cases and 290,000–650,000 deaths (Iuliano et al., 2018). Influenza vaccination is the most effective measure to prevent influenza infection, which is recommended for pregnant women, children, the elderly, health care workers, and individuals with specific chronic diseases in priority. The most commonly used influenza vaccine is trivalent inactivated influenza virus split vaccine, containing two influenza A antigens (A/H1N1 and A/H3N2) and one influenza B antigen (B/Victoria or B/Yamagata). Some studies have suggested that 0.3%–10% of healthy individuals with influenza vaccination still failed to elicit protective antibodies (Narang et al., 2018; Soedjatmiko et al., 2018). Several host factors play important roles in the regulation of humoral response to influenza vaccine, such as age, gender, health status, pre-existing immunity, and genetic factors (Dhakal and Klein, 2019). Among the genetic factors, many single nucleotide polymorphisms (SNPs) in human leucocyte antigen (HLA), cytokine, and cytokine receptor-related genes have been confirmed to be significantly associated with humoral response to influenza vaccine (Poland et al., 2008; Franco et al., 2013).

Leptin (LEP), an adipocyte-derived hormone, is known for its ability to regulate energy metabolism and immune response (Francisco et al., 2018). The immune-modulating effect of leptin starts with its binding to the leptin receptor (LEPR) expressed on the membrane of immune cells. It is reported that leptin regulates the proliferation and reactivity of T cells by activating the JAK/STAT pathway to influence cellular immunity (Kim et al., 2010). Moreover, leptin participates in the humoral immune response by up-regulation the expressing of TNF-α, IL-6, and IL-10 (Agrawal et al., 2011). In addition, peroxisome proliferator activated receptor gamma (PPARG) coordinates with leptin and also plays a role in adipocyte differentiation and inflammatory response in protein interaction network (Joffe and Houghton, 2016). There are many SNPs in the promoter, intron, and exon regions of *LEP*, *LEPR*, and *PPARG*, which might regulate related genes transcription, splicing, and translation. Previous studies have reported that some leptin-related gene polymorphisms were associated with the influenza antibody levels to influenza vaccine in elderly Caucasians (Ovsyannikova et al., 2014). Nevertheless, the association between SNPs in *LEP*, *LEPR*, and *PPARG* and influenza vaccine-induced immune response has not been revealed in the Chinese Han population.

In this study, we detected 11 tag SNPs in 592 subjects and hypothesized that certain SNPs may explain the poor immunogenicity of influenza vaccine. This study aimed to elucidate the association of SNPs in *LEP*, *LEPR*, and *PPARG* with humoral immune response to influenza vaccine in the Chinese Han population.

## Materials and Methods

### Study Design and Subjects

**FIGURE 1:**
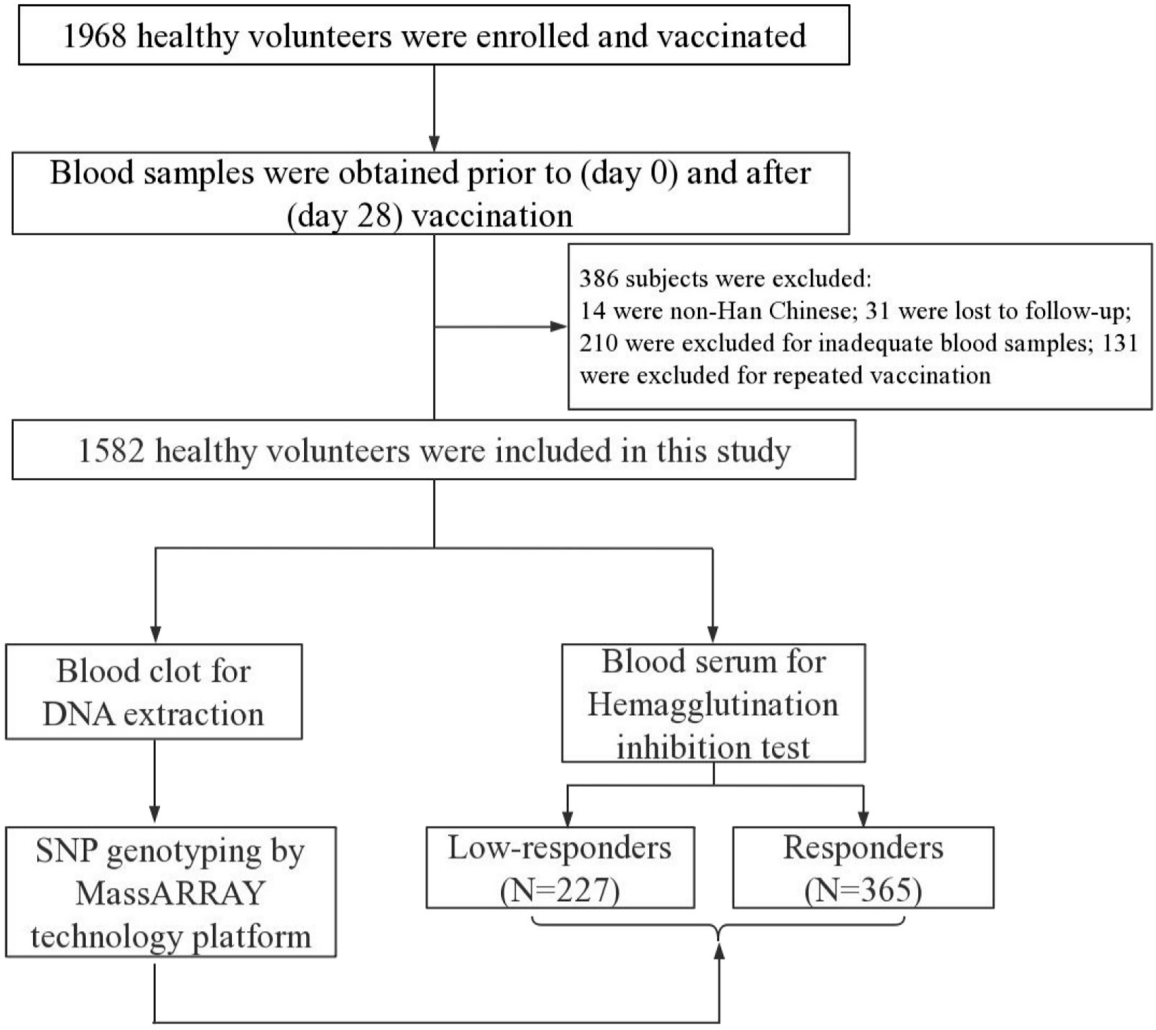
Flow diagram of study.

In total, 1968 healthy volunteers were consecutively recruited in this study at Xinjiang Uygur Autonomous Region and Yunnan Province, China, from September 2009 to September 2019. All subjects were only vaccinated one intramuscular dose of trivalent inactivated seasonal influenza vaccine (TIV, Fluarix, 0.50 ml), which was manufactured according to the World Health Organization (WHO) recommendation for the northern hemisphere. Subsequently, 386 subjects were excluded for non-Han Chinese, loss to follow-up, inadequate blood samples, and repeat vaccinations. The flow diagram of this study was shown in Figure 1. We finally selected 592 individuals according to the seroconversion to A/H1N1, A/H3N2, and B vaccine components, including 227 low-responders (achieved seroconversion to none of vaccine components) and 365 responders (achieved seroconversion to all vaccine components). A case-control study design was conducted to analyze the association of SNPs in *LEP*, *LEPR,* and *PPARG* with humoral response to influenza vaccine in the Chinese Han population. Questionnaires were collected to obtain personal information, such as gender, age, vaccine type, vaccination history, etc. Peripheral venous blood samples were collected prior to (day 0) and after (day 28) vaccination and stored at −30°C.

### SNP Selection and Genotyping

Eleven tag SNPs in *LEP*, *LEPR,* and *PPARG* were selected using dbSNP database (https://www.ncbi.nlm.nih.gov/snp/) and SNPinfo Web Server (https://manticore.niehs.nih.gov/) with minor allele frequency (MAF) > 0.05 in the Chinese Han Beijing (CHB) population and *r* ^2^ > 0.8, including rs2167270 and rs11761556 in *LEP*, rs1327118, rs7602, rs1137101, rs1938489, rs6673591, rs1137100, and rs13306523 in *LEPR*, and rs796313 and rs17793951 in *PPARG* (Supplementary Table 1). Genome DNA was extracted using an AxyPrep Blood Genomic DNA Miniprep kit (Axygen, Union City, California, United States) according to the manufacturer’s instructions. Genotyping of candidate SNPs was conducted using the MassARRAY technology platform (Sequenom, San Diego, California, United States) and implemented by BioMiao Biological Technology (Beijing, China). As shown in Supplementary Table 2, the call rates for 11 tag SNPs in *LEP*, *LEPR,* and *PPARG* were all higher than 98.0%. Additionally, we used HaploReg (V.4.1, https://pubs.broadinstitute.org/mammals/haploreg/haploreg.php) to explore protein binding annotations of SNPs.

### Influenza Hemagglutination Inhibition (HAI) Assay

The HAI assays were used to measure sera antibody titers with 1% solution of turkey red blood cells against influenza vaccine strains according to standard methods described in the WHO guideline (World Health Organization, 2011World Health Organization, 2011). The vaccine strains recommended by WHO for the northern hemisphere from 2009–2010 to 2019–2020 flu seasons are summarized in Supplementary Table 3. Seroconversion was defined as either post-vaccination HAI titer ≥40 if pre-vaccination HAI titer <10 or at least fourfold increase if pre-vaccination HAI titer ≥10. Moreover, seroconversion factors (SCFs) were considered as the fold rises of geometric mean titer (GMT) of HAI antibody titers post-vaccination.

### Statistical Analysis

The Hardy-Weinberg equilibrium (HWE) test was conducted to assess the genotype frequencies of SNPs among subjects. Continuous variables with normal distribution through the Kolmogorov-Smirnov test were described as the‾x ± s and analyzed by One-way ANOVA (analysis of variance). Categorical data was summarized as frequencies (percentages) and compared using χ^2^ test or Fisher’s exact test appropriately. Associations of tag SNPs with immune response to influenza vaccine were analyzed using univariate and multivariate logistic regression models adjusted for gender and age in additive genetic model and dominant genetic model, and the odds ratios (95% CIs) were also estimated. We also conducted a stratified analysis to explore this association in females or males, separately. The pairwise linkage disequilibrium (LD) and haplotype analysis were performed using SNPStats (http://bioinfo.iconcologia.net/SNPStats). Gene-gene interactions were calculated with the GMDR program (V.0.7, http://sourceforge.net/projects/gmdr/). All analyses were performed by the SPSS software (V.25.0) and GraphPad Prism software (V.6.01), and the significance level was set at *p* < 0.05 with two-tailed. When we compared the genotypic frequencies of 11 SNPs between the two groups, the significance level was set at *p* < 0.0045 (0.05/11) after the Bonferroni corrections.

## Results

### Characteristics of Subjects

To investigate the association of SNPs in *LEP*, *LEPR,* and *PPARG* with humoral response to influenza vaccine in the Chinese Han population, 592 subjects were recruited in this study including 227 low-responders and 365 responders. The general characteristics were shown in Supplementary Table 4. There was no significant difference in gender and age between the two groups.

### Comparison of Genotypic Frequencies of *LEP*, *LEPR,* and *PPARG* Between Low-Responders and Responders

A total of 11 SNPs in *LEP*, *LEPR,* and *PPARG* were genotyped, and genotype distribution of all SNPs in the 592 subjects conformed to Hardy-Weinberg equilibrium (*p* > 0.05). The information regarding the genotyped SNPs was described in detail in Supplementary Table 1, in terms of chromosome positions, consequences, alleles, and minor allele frequencies. The MAFs of all SNPs were in accordance with those in the CHB population, which were reported by the 1000 Genome Project (phase 3). Furthermore, additive genetic model and dominant genetic model were performed to investigate the association between 11 tag SNPs and humoral response to influenza vaccine. Since few subjects were carrying *PPARG* rs17793951 GG genotype, we only compared the genotypic frequencies of AA and AG between the two groups in the additive genetic model. The results indicated the genotypic frequencies of *PPARG* rs17793951 AG in low-responders was higher than that in responders (*p* = 0.001), and the multivariate logistic regression analysis showed that AG genotype was correlated with a higher risk of low responsiveness to influenza vaccine adjusted for gender and age (OR = 2.94, 95% CI = 1.67–5.19). This difference was still significant after the Bonferroni correction. In dominant genetic model analysis, the *PPARG* rs17793951 AG + GG genotype was related with lower response to influenza vaccine in both univariate logistic regression analysis (OR = 2.71, 95% CI = 1.56–4.75) and multivariate logistic regression analysis (OR = 2.81, 95% CI = 1.61–4.92). However, no significant association of tag SNPs in *LEP* and *LEPR* with low responsiveness to influenza vaccine was found in our study (Table 1).

**TABLE 1:** The genotypic frequencies and odds ratios of SNPs in *LEP*, *LEPR,* and *PPARG* between the two groups in additive genetic model and dominant genetic model.

Gene	SNP ID	Genotype	Low-responders N^a^ (%)	Responders N^a^ (%)	OR (95% CI)^b^	OR (95% CI)^c^	*P*_*HWE*_
*LEP*	rs2167270	GG	159 (71.0)	232 (64.3)	1.00 (reference)	1.00 (reference)	0.737
		AG	58 (25.9)	118 (32.7)	0.72 (0.49–1.04)	0.72 (0.50–1.05)	
		AA	7 (3.1)	11 (3.0)	0.93 (0.35–2.45)	1.10 (0.41–2.96)	
		AG + AA	65 (29.0)	129 (35.7)	0.74 (0.51–1.05)	0.75 (0.52–1.08)	
	rs11761556	AA	129 (57.1)	197 (54.6)	1.00 (reference)	1.00 (reference)	0.612
		AC	88 (38.9)	138 (38.2)	0.97 (0.69–1.38)	0.98 (0.69–1.39)	
		CC	9 (4.0)	26 (7.2)	0.53 (0.24–1.17)	0.58 (0.26–1.28)	
		AC + CC	97 (42.9)	164 (45.4)	0.90 (0.65–1.26)	0.92 (0.65–1.28)	
*LEPR*	rs1327118	CC	160 (70.8)	270 (74.8)	1.00 (reference)	1.00 (reference)	0.956
		CG	63 (27.9)	82 (22.7)	1.30 (0.89–1.90)	1.35 (0.92–1.98)	
		GG	3 (1.3)	9 (2.5)	0.56 (0.15–2.11)	0.58 (0.15–2.20)	
		CG + GG	66 (29.2)	91 (25.2)	1.22 (0.84–1.78)	1.27 (0.87–1.85)	
	rs7602	GG	165 (73.0)	255 (70.2)	1.00 (reference)	1.00 (reference)	0.946
		GA	56 (24.8)	99 (27.3)	0.87 (0.60–1.28)	0.89 (0.61–1.31)	
		AA	5 (2.2)	9 (2.5)	0.86 (0.28–2.61)	0.86 (0.28–2.64)	
		GA + AA	61 (27.0)	108 (29.8)	0.87 (0.60–1.26)	0.89 (0.61–1.29)	
	rs1137101	GG	167 (74.2)	272 (75.6)	1.00 (reference)	1.00 (reference)	0.649
		GA	56 (24.9)	81 (22.5)	1.13 (0.76–1.67)	1.13 (0.76–1.67)	
		AA	2 (0.9)	7 (1.9)	0.47 (0.10–2.27)	0.49 (0.10–2.41)	
		GA + AA	58 (25.8)	88 (24.4)	1.07 (0.73–1.58)	1.08 (0.73–1.59)	
	rs1938489	AA	198 (88.0)	325 (89.8)	1.00 (reference)	1.00 (reference)	0.528
		AG	26 (11.6)	37 (10.2)	1.15 (0.68–1.96)	1.16 (0.68–1.98)	
		GG	1 (0.4)	0 (0.0)	-	-	
		AG + GG	27 (12.0)	37 (10.2)	1.20 (0.71–2.03)	1.20 (0.71–2.05)	
	rs6673591	GG	172 (76.4)	278 (76.8)	1.00 (reference)	1.00 (reference)	0.482
		GA	51 (22.7)	79 (21.8)	1.04 (0.70–1.56)	1.04 (0.70–1.56)	
		AA	2 (0.9)	5 (1.4)	0.65 (0.12–3.37)	0.71 (0.14–3.73)	
		GA + AA	53 (23.6)	84 (23.2)	1.02 (0.69–1.51)	1.02 (0.69–1.52)	
	rs1137100	GG	142 (62.8)	234 (64.8)	1.00 (reference)	1.00 (reference)	0.742
		GA	73 (32.3)	113 (31.3)	1.07 (0.74–1.53)	1.07 (0.75–1.54)	
		AA	11 (4.9)	14 (3.9)	1.30 (0.57–2.93)	1.33 (0.58–3.02)	
		GA + AA	84 (37.2)	127 (35.2)	1.09 (0.77–1.54)	1.10 (0.78–1.56)	
	rs13306523	CC	209 (92.1)	339 (93.4)	1.00 (reference)	1.00 (reference)	0.370
		CT	18 (7.9)	24 (6.6)	1.22 (0.65–2.30)	1.22 (0.64–2.32)	
		TT	0 (0.0)	0 (0.0)	-	-	
		CT + TT	18 (7.9)	24 (6.6)	1.22 (0.65–2.30)	1.22 (0.64–2.32)	
*PPARG*	rs796313	GG	80 (35.6)	121 (33.2)	1.00 (reference)	1.00 (reference)	0.275
		GT	114 (50.7)	183 (50.3)	0.94 (0.65–1.36)	0.91 (0.62–1.31)	
		TT	31 (13.7)	60 (16.5)	0.78 (0.47–1.31)	0.78 (0.46–1.31)	
		GT + TT	145 (64.4)	243 (66.8)	0.90 (0.64–1.28)	0.87 (0.61–1.24)	
	rs17793951	AA	190 (84.4)	340 (93.7)	1.00 (reference)	1.00 (reference)	0.678
		AG	35 (15.6)	22 (6.1)	2.85 (1.62–4.99)^*^	2.94 (1.67–5.19)^*^	
		GG	0 (0.0)	1 (0.3)	-	-	
		AG + GG	35 (15.6)	23 (6.3)	2.72 (1.56–4.75)	2.81 (1.61–4.92)	

* The *p* value (*p* = 0.001) of trend analysis was statistically significant after Bonferroni correction.

a The number may not equal to the total number due to missing data.

b Unadjusted any variables.

c Adjusted for age and gender.

P_HWE_ was the value of Hardy-Weinberg equilibrium test.

### *PPARG* rs17793951 AG + GG Carriers had Lower Antibody Fold Rises

**FIGURE 2:**
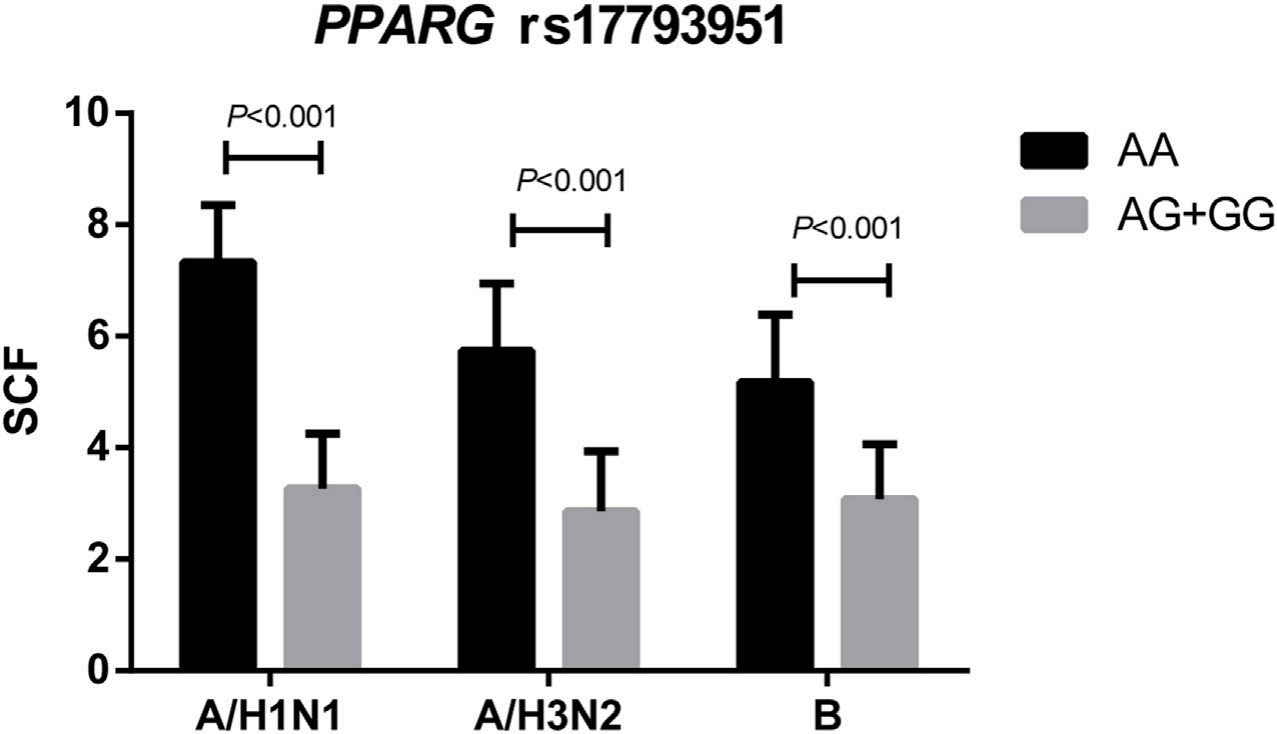
Comparison the SCFs of different vaccine components between *PPARG* rs17793951 AA carriers and AG + GG carriers.

The comparison of SCFs of different vaccine components between *PPARG* rs17793951 AA carriers and AG + GG carriers was shown in Figure 2. The antibody fold rises of *PPARG* rs17793951 AG + GG genotype carriers were significantly lower than that of AA genotype carriers against all three components (*p* < 0.001). The specific SCF values were shown in Supplementary Table 5.

### Stratification Analysis of the Association Between Responsiveness to Influenza Vaccine and *LEPR*/*PPARG* Polymorphisms

The stratified analysis found that *LEPR* rs6673591 GA + AA genotype was related to low responsiveness to influenza vaccine in males (OR = 1.96, 95% CI = 1.05–3.67). However, the contrary correlation was found in females even if without statistical significance (OR = 0.63, 95% CI = 0.37–1.08). We also found that the genotypic frequencies of *PPARG* rs17793951 AG + GG in low-responders were higher than that in responders both in males and females, but it was significant only in females (Table 2).

**TABLE 2:** Association of SNPs in *LEPR* and *PPARG* with influenza vaccine-induced humoral responses stratified by gender.

Gene	SNP ID	Genotype	Female (N = 359)			Male (N = 233)		
			Low-responders N^a^ (%)	Responders N^a^ (%)	OR (95%CI)^b^	Low-responders N* (%)	Responders N* (%)	OR (95%CI)^b^
*LEPR*	rs6673591	GG	110 (81.5)	166 (74.4)	1.00 (reference)	62 (68.9)	112 (80.6)	1.00 (reference)
		GA + AA	25 (18.5)	57 (25.6)	0.63 (0.37–1.08)	28 (31.1)	27 (19.4)	1.96 (1.05–3.67)
*PPARG*	rs17793951	AA	112 (83.0)	209 (93.7)	1.00 (reference)	78 (86.7)	131 (93.6)	1.00 (reference)
		AG + GG	23 (17.0)	14 (6.3)	3.28 (1.61–6.67)	12 (13.3)	9 (6.4)	2.18 (0.87–5.46)

a The number may not equal to the total number due to missing data.

b Adjusted for age.

### LD and Haplotype Analysis of SNPs in *LEP*, *LEPR,* and *PPARG*

The linkage disequilibrium (LD) structures of two SNPs in *LEP*, seven SNPs in *LEPR,* and two SNPs in *PPARG* were shown in Supplementary Tables 6–8, and they were not in LD. Haplotypes in *LEP* were not correlated with response to influenza vaccine (Supplementary Table 9). Furthermore, compared with haplotype CGGAGGC, we found that the haplotype CAAAAAC in the *LEPR* was positively associated with responsiveness to influenza vaccine (*p* = 0.010, OR = 0.34, 95% CI = 0.15–0.77, Table 3). Haplotype blocks were also built between *PPARG* rs796313 and rs17793951, and four haplotypes were identified (Table 4). Compared with haplotype GA, the uncommon haplotype TG was associated with low responsiveness to influenza vaccine (*p* = 0.002, OR = 3.85, 95% CI = 1.67–9.09). Moreover, no gene-gene interaction among 11 SNPs of *LEP*, *LEPR,* and *PPARG* in the GMDR method was found (Supplementary Table 10).

**TABLE 3:** Association between haplotypes of *LEPR* and low responsiveness to influenza vaccine.

*LEPR* Haplotype	SNP^a^							Frequency			OR (95%CI)	*P*
	1	2	3	4	5	6	7	Total	Low-responders	Responders		
1	C	G	G	A	G	G	C	0.63	0.65	0.63	1.00 (reference)	-
2	G	G	G	A	G	G	C	0.06	0.05	0.06	0.95 (0.56–1.64)	0.860
3	C	A	A	A	A	A	C	0.04	0.02	0.06	0.34 (0.15–0.77)	0.010
4	C	G	A	A	A	A	C	0.04	0.05	0.03	1.54 (0.76–3.03)	0.230
5	G	A	G	A	G	G	C	0.03	0.03	0.04	0.74 (0.35–1.59)	0.440
6	C	G	G	G	G	A	C	0.03	0.02	0.03	0.66 (0.27–1.64)	0.370
7	C	A	G	A	G	G	C	0.03	0.02	0.03	0.85 (0.36–2.00)	0.720
8	C	G	G	A	G	G	T	0.02	0.02	0.02	1.49 (0.60–3.70)	0.390
9	G	G	A	A	A	A	C	0.02	0.03	0.01	2.13 (0.76–5.88)	0.150
10	C	A	G	A	G	A	C	0.02	0.02	0.01	1.79 (0.68–4.76)	0.240
11	G	A	A	A	A	A	C	0.01	0.02	0.01	3.85 (0.68–20.00)	0.130
12	C	A	G	A	G	G	T	0.01	0.01	0.01	1.12 (0.34–3.85)	0.850
13	C	G	A	A	G	G	C	0.01	0.01	0.01	0.93 (0.25–3.45)	0.910
14	G	G	G	G	G	A	C	0.01	0.01	0.01	0.60 (0.17–2.08)	0.420
Rare^b^	-	-	-	-	-	-	-	-	-	-	-	-

SNP^a^: 1-rs1327118, 2-rs7602, 3-rs1137101, 4-rs1938489, 5-rs6673591, 6-rs1137100, and 7-rs13306523.

Rare^b^: Haplotypes with frequencies <0.01.

**TABLE 4:** Association between haplotypes of *PPARG* and low responsiveness to influenza vaccine.

*PPARG* Haplotype	SNP			Frequency		OR (95% CI)	*P*
	rs796313	rs17793951	Total	Low-responders	Responders		
1	G	A	0.57	0.59	0.56	1.00 (reference)	-
2	T	A	0.38	0.33	0.40	0.78 (0.60–1.02)	0.073
3	T	G	0.03	0.06	0.01	3.85 (1.67–9.09)	0.002
4	G	G	0.02	0.02	0.02	1.08 (0.36–3.23)	0.900

## Discussion

To investigate the roles of *LEP*, *LEPR,* and *PPARG* in the humoral immune response to influenza vaccine, we conducted an association study to compare the genotypic frequencies of 11 tag SNPs in these genes between the two groups in the Chinese Han population. The most significant result was that *PPARG* rs17793951 AG + GG genotype was associated with low responsiveness to influenza vaccine, and we also observed that *LEPR* rs6673591 GA + AA genotype was correlated with low responsiveness to influenza vaccine only in males by stratified analysis.

Leptin is a protein hormone (16 kDa) secreted by adipocytes. It directly acts on CD4^+^ T helper cells, CD8^+^ cytotoxic T cells, and B cells by interacting with the membrane-bound leptin receptor (Papathanassoglou et al., 2006). A recent study reported that increased concentrations of leptin may help maintain the naive T cell pool in the elderly (Chen et al., 2010). Evidence also showed that leptin signaling regulates B cell homeostasis through activating Bcl-2 and cyclin D1 (Lam et al., 2010). Although previous studies suggested that leptin played important roles in regulation specific-IgG antibody levels of influenza vaccine and clearance of influenza virus (Zhang et al., 2013; Frasca et al., 2020), we didn’t find significant associations between tag SNPs in *LEP* and *LEPR* and antibody response to influenza vaccine, which was in agreement with a previous report (Ovsyannikova et al., 2014). In general, there were few studies focused on the relationship between leptin and influenza vaccines, and the underlying mechanisms were still unclear.

As we know, PPARG is a lipid-activated nuclear receptor expressed on the surface of macrophages, dendritic cells, and regulatory T cells (Odegaard et al., 2007; Tuna et al., 2014; Khare et al., 2015). A recent study has demonstrated that three SNPs in *PPARG* were significantly related to the baseline level of immunomodulator, Vitamin D, and the authors presumed that PPARG and Vitamin D receptors may interact with each other and participate in immune response to influenza vaccine (Sadarangani et al., 2016). Furthermore, PPARG help to restrict pulmonary inflammation and promote recovery from influenza virus infection (Huang et al., 2019). These studies suggested that PPARG may play a role in immune response to influenza virus or vaccine. Indeed, our study found that *PPARG* rs17793951 was associated with immune response to influenza vaccine in additive genetic model and dominant genetic model. The risk of low responsiveness to influenza vaccine in *PPARG* rs17793951 AG + GG genotype was almost 3 times higher than that of AA genotype in multivariate logistic regression analysis. Subjects carrying AG + GG genotype had a lower fold-rise of antibody titer to all vaccine components. Similarly, an American study (Ovsyannikova et al., 2014) used the linear correlation methods to explore the association of leptin-related genes variants with the antibody response to influenza vaccine in 50–80-year-old Caucasians, and they found that *PPARG* rs17793951G minor allele was associated with the A/H1N1 HAI antibody titers. Although there were some different aspects on subjects’ populations and study design between this research and our study, the results both showed that *PPARG* rs17793951 variants might play an important role in immune response to influenza vaccine. Unfortunately, there was still no research to demonstrate the exact mechanism of this association. What’s more, the rs17793951 was located in intron two of *PPARG* and could bind to CCCTC-binding factor (CTCF) in the functional annotations of HaploReg V.4.1. Specifically, CTCF is a transcription factor and has many effects on chromosome segregation and regulation of genes expression as an insulator (Kang and Lee, 2021; Pongubala and Murre, 2021). Therefore, we hypothesized that the rs17793951 A to G change may suppress *PPARG* transcription by enhancing the binding ability of this site to CTCF, resulting in a downregulation of immune response consequently. More research is needed to verify the association of *PPARG* rs17793951 with humoral response to influenza vaccine and reveal its physiological mechanisms.

Gender is an important factor affecting immune response to influenza vaccine. Stratified analysis implicated that *LEPR* rs6673591 GA + AA genotype had an approximately doubled risk of low responsiveness compared with AA genotype in males. On the contrary, *LEPR* rs6673591 GA + AA genotype in females tends to correlate with higher antibody response, but the difference was not statistically significant. Moreover, *PPARG* rs17793951 AG + GG genotype was associated with a higher risk of low responsiveness to influenza vaccine in males and females, but this effect was significant only in females. An article also reported gender difference in the association of genetic polymorphisms with immune response to Japanese encephalitis vaccine (Yao et al., 2020). We speculated that some immune-suppressive behaviors more commonly in men may mask the correlation of genotypes with response to influenza vaccine, such as smoking (Godoy et al., 2018). Additionally, women have higher concentrations of leptin and estrogen normally (Thomas et al., 2000), with up-regulation of immune response, thereby there were modification effects of gender on the association of SNPs with humoral response to influenza vaccine. It is worthy to explore the mechanisms of gender difference on the association of SNPs with antibody response to influenza vaccine.

Our study demonstrated that the haplotype CAAAAAC in the *LEPR* gene composed of seven tag SNPs was positively correlated with vaccine response compared with the CGGAGGC haplotype, which was a joint contribution of rs7602 in 3′UTR, rs6673591 in intron, rs1137101 and rs1137100 in exons, although no significant association was found in single SNP analysis. This discordance may be explained by the fact that vaccine response is a complex process involving multiple factors and each of the SNPs has a tiny influence on immune response. Moreover, when haplotypes were constructed with *PPARG* rs17793951 and rs796313 SNPs, we found a more significant association between TG haplotype with poor responsiveness to influenza vaccine, which was consistent with single SNP analysis. The results further suggested that *PPARG* rs17793951 polymorphism played an important role in immune response to influenza vaccine*.* Additional studies are required to verify our hypotheses in different populations.

As far as we know, this is the first research to examine the association between SNPs in *LEP*, *LEPR,* and *PPARG* and the humoral response of A/H1N1, A/H3N2, and B vaccine components in the Chinese Han population. There are still several limitations in our study. A relatively small sample of subjects was used in our study. However, our subjects were carefully selected based on seroconversion to all or none of vaccine components with sufficient statistical power, and the MAFs of tag SNPs were in accordance with that of the 1000 Genome Project (phase 3). Although we adjusted for the known influencing factors to evaluate this association, such as age and gender, there may exist other uncollected factors affecting immune response to influenza vaccine. In addition, our study only assessed the antibody level on the 28th day post-vaccination when it reached a peak, yet antibody level will change over time. Therefore, future studies are required to investigate this association through longer-term observation and provide more sufficiently theoretical evidence for the development of universal influenza vaccines.

## Conclusion

The present study demonstrated the association between *PPARG* rs17793951 AG + GG genotype and low responsiveness to influenza vaccine in the Chinese Han population.

## Data Availability

The original contributions presented in the study are included in the article/Supplementary Material, further inquiries can be directed to the corresponding authors.
