# Supplementary material for: Association of Single Nucleotide Polymorphisms in LEP, LEPR, and PPARG With Humoral Immune Response to Influenza Vaccine
Source: Front Genet. 2021 Oct 22;12:725538. doi: 10.3389/fgene.2021.725538 (PMC8569447; doi:10.3389/fgene.2021.725538)
Supplement: Supplementary file 1 [file DataSheet1.doc]

## SUPPLEMENTARY DATA

**Supplementary Table 1.** The detail information of 11 tag SNPs in the *LEP*, *LEPR* and *PPARG*.

| **Gene** | **SNP ^a^** | **Chromosome position ^b^** | **Consequence** | **Allele** | **MAF** | **CHB MAF** |
| --- | --- | --- | --- | --- | --- | --- |
| *LEP* | rs2167270 | 7:128241296 | 5 Prime UTR Variant | G/A | 0.18 | 0.21 |
| *LEP* | rs11761556 | 7:128257016 | 3 Prime UTR Variant | A/C | 0.25 | 0.29 |
| *LEPR* | rs1327118 | 1:65419886 | 2KB Upstream Variant | C/G | 0.14 | 0.13 |
| *LEPR* | rs7602 | 1:65432268 | Intron Variant | G/A | 0.16 | 0.14 |
| *LEPR* | rs1137101 | 1:65592830 | Missense Variant | G/A | 0.13 | 0.13 |
| *LEPR* | rs1938489 | 1:65582939 | Intron Variant | A/G | 0.06 | 0.05 |
| *LEPR* | rs6673591 | 1:65582706 | Intron Variant | G/A | 0.12 | 0.11 |
| *LEPR* | rs1137100 | 1:65570758 | Missense Variant | G/A | 0.20 | 0.19 |
| *LEPR* | rs13306523 | 1:65425307 | 5 Prime UTR Variant | C/T | 0.04 | 0.06 |
| *PPARG* | rs796313 | 3:12408029 | Intron Variant | G/T | 0.41 | 0.45 |
| *PPARG* | rs17793951 | 3:12329238 | Intron Variant | A/G | 0.05 | 0.05 |

^a^ According to the dbSNP database.

^b^ The SNP chromosome positions and consequences are on the NCBI human genome.

**Supplementary Table 2.** The call rates of SNPs for genotyping.

| **Gene** | **SNP** | **Call rates (%)** |
| --- | --- | --- |
| *LEP* | rs2167270 | 98.82 |
|  | rs11761556 | 99.16 |
| *LEPR* | rs1327118 | 99.16 |
|  | rs7602 | 99.49 |
|  | rs1137101 | 98.82 |
|  | rs1938489 | 99.16 |
|  | rs6673591 | 99.16 |
|  | rs1137100 | 99.16 |
|  | rs13306523 | 99.66 |
| *PPARG* | rs796313 | 99.49 |
|  | rs17793951 | 99.32 |

**Supplementary Table 3.** Vaccine strains recommended by WHO for the northern hemisphere.

| **Flu season** | **A/H1N1** | **A/H3N2** | **B/Victoria or B/Yamagata** |
| --- | --- | --- | --- |
| 2009-2010 | A/Brisbane/59/2007 (H1N1) | A/Brisbane/10/2007 (H3N2) | B/Brisbane/60/2008(B/Victoria) |
| 2010-2011 | A/California/7/2009 (H1N1) | A/Perth/16/2009 (H3N2) | B/Brisbane/60/2008(B/Victoria) |
| 2011-2012 | A/California/7/2009 (H1N1) | A/Perth/16/2009 (H3N2) | B/Brisbane/60/2008(B/Victoria) |
| 2012-2013 | A/California/7/2009 (H1N1) | A/Victoria/361/2011 (H3N2) | B/Wisconsin/1/2010(B/Yamagata) |
| 2013-2014 | A/California/7/2009 (H1N1) | A/Texas/50/2012 (H3N2) | B/Massachusetts/2/2012(B/Yamagata) |
| 2014-2015 | A/California/7/2009 (H1N1) | A/Texas/50/2012 (H3N2) | B/Massachusetts/2/2012(B/Yamagata) |
| 2015-2016 | A/California/7/2009 (H1N1) | A/Switzerland/9715293/2013 (H3N2) | B/Phuket/3073/2013(B/Yamagata) |
| 2016-2017 | A/California/7/2009 (H1N1) | A/Hong Kong/4801/2014 (H3N2) | B/Brisbane/60/2008(B/Victoria) |
| 2017-2018 | A/Michigan/45/2015 (H1N1) | A/Hong Kong/4801/2014 (H3N2) | B/Brisbane/60/2008(B/Victoria) |
| 2018-2019 | A/Michigan/45/2015 (H1N1) | A/Singapore/INFIMH-16-0019/2016 (H3N2) | B/Colorado/06/2017 (B/Victoria) |
| 2019-2020 | A/Brisbane/02/2018 (H1N1) | A/Kansas/14/2017 (H3N2) | B/Colorado/06/2017 (B/Victoria) |

**Supplementary Table 4.** General characteristics of subjects with low responsiveness and response to influenza vaccine.

| **Variable** | **Low-responders**  **N (%)** | **Responders**  **N (%)** | **χ^2^** | ***P*** |
| --- | --- | --- | --- | --- |
| Gender |  |  | 0.08 | 0.774 |
| Female | 136(59.9) | 223(61.1) |
| Male | 91(40.1) | 142(38.9) |
| Age (years) |  |  | 6.21 | 0.102 |
| <5 | 54(23.8) | 84(23.0) |
| 5-17 | 16 (7.1) | 45(12.3) |
| 18-64 | 107(47.1) | 176(48.3) |
| ≥65 | 50(22.0) | 60(16.4) |

**Supplementary Table 5.** The specific SCF values of different vaccine components between *PPARG* rs17793951 AA carriers and AG+GG carriers.

| **Vaccine component** | **AA (N=530)** | **AG+GG (N=58)** |
| --- | --- | --- |
| A/H1N1 | 7.33±1.02 | 3.27±0.98 |
| A/H3N2 | 5.74±1.21 | 2.86±1.08 |
| B | 5.18±1.21 | 3.08±0.98 |

Seroconversion factor (SCF) = the GMT of antibody HAI titers post-vaccination /the GMT of antibody HAI titers pre-vaccination.

**Supplementary Table 6.** The linkage disequilibrium coefficients between two SNPs of *LEP*.

|  | **rs2167270** | **rs11761556** |
| --- | --- | --- |
| rs2167270 | - | 0.808 |
| rs11761556 | 0.429 | - |

Values on the left of “-” are r^2^ and on the right are Lewontin’s D’ coefficients.

**Supplementary Table 7.** The linkage disequilibrium coefficients among seven SNPs of *LEPR*.

|  | **rs1327118** | **rs7602** | **rs1137101** | **rs1938489** | **rs6673591** | **rs1137100** | **rs13306523** |
| --- | --- | --- | --- | --- | --- | --- | --- |
| rs1327118 | - | 0.178 | 0.109 | 0.095 | 0.141 | 0.175 | 0.987 |
| rs7602 | 0.029 | - | 0.319 | 0.006 | 0.346 | 0.412 | 0.197 |
| rs1137101 | 0.011 | 0.084 | - | 0.182 | 0.908 | 0.893 | 0.986 |
| rs1938489 | 0.003 | <0.001 | <0.001 | - | 0.990 | 0.999 | 0.041 |
| rs6673591 | 0.016 | 0.091 | 0.755 | 0.008 | - | 1.000 | 0.984 |
| rs1137100 | 0.020 | 0.124 | 0.484 | 0.232 | 0.552 | - | 0.991 |
| rs13306523 | 0.006 | 0.008 | 0.005 | 0.001 | 0.005 | 0.009 | - |

Values on the left of “-” are r^2^ and on the right are Lewontin’s D’ coefficients.

**Supplementary Table 8.** The linkage disequilibrium coefficients between two SNPs of *PPARG*.

|  | **rs796313** | **rs17793951** |
| --- | --- | --- |
| rs796313 | - | 0.295 |
| rs17793951 | 0.007 | - |

Values on the left of “-” are r^2^ and on the right are Lewontin’s D’ coefficients.

**Supplementary Table 9.** Association between haplotypes of *LEP* and low responsiveness to influenza vaccine.

| ***LEP***  **Haplotype** | **SNP** | | **Frequency** | | | **OR (95% CI)** | ***P*** |
| --- | --- | --- | --- | --- | --- | --- | --- |
|  | **rs2167270** | **rs11761556** | **Total** | **Low-responders** | **Responders** |  |  |
| 1 | G | A | 0.72 | 0.74 | 0.71 | 1.00 | - |
| 2 | A | C | 0.15 | 0.13 | 0.17 | 0.79(0.56-1.12) | 0.190 |
| 3 | G | C | 0.10 | 0.10 | 0.10 | 1.04(0.68-1.59) | 0.870 |
| 4 | A | A | 0.03 | 0.03 | 0.03 | 1.04(0.48-2.27) | 0.920 |

**Supplementary Table 10.** Gene-gene interactions among *LEP*, *LEPR* and *PPARG* genetic variants.

| **Model** | **Training balance accuracy** | **Testing balance accuracy** | ***P*** | **Cross-validation consistency** |
| --- | --- | --- | --- | --- |
| rs17793951 | 0.547 | 0.545 | 0.011 | 10/10 |
| rs1327118 rs17793951 | 0.571 | 0.530 | 0.172 | 8/10 |
| rs1137101 rs796313 rs17793951 | 0.597 | 0.559 | 0.172 | 8/10 |
| rs1327118 rs1137101 rs796313 rs17793951 | 0.614 | 0.540 | 0.172 | 5/10 |
| rs1327118 rs7602 rs1137101 rs11761556 rs796313 | 0.635 | 0.520 | 0.377 | 2/10 |
| rs1327118 rs7602 rs1137101 rs1137100 rs796313 rs17793951 | 0.663 | 0.544 | 0.055 | 4/10 |
| rs1327118 rs7602 rs1137101 rs1938489 rs11761556 rs796313 rs17793951 | 0.685 | 0.552 | 0.055 | 5/10 |
| rs1327118 rs2167270 rs7602 rs1137101 rs1137100 rs11761556 rs796313 rs17793951 | 0.706 | 0.554 | 0.172 | 9/10 |
| rs1327118 rs2167270 rs7602 rs1137101 rs1938489 rs1137100 rs11761556 rs796313 rs17793951 | 0.721 | 0.566 | 0.055 | 8/10 |

*P* was calculated by GMDR V.0.7 software.
